# Post-Burn and Surgical Scar Reconstruction with Tissue Expanders: Review of the Literature and Our Local Experience

**DOI:** 10.3390/reports7010001

**Published:** 2023-12-21

**Authors:** Ziyad I. Alharbi, Leena H. Moshref, Rahaf E. Badr, Ola A. Zahran, Maan T. Almaghrabi, Sherif F. Khamis

**Affiliations:** 1 Department of Plastic Surgery, Dr. Soliman Fakeeh Hospital, Jeddah P.O. Box 23323, Saudi Arabia; maanfm@gmail.com (M.T.A.); sfkhamis@fakeeh.care (S.F.K.); 2 Plastic Surgery, Dr. Soliman Fakeeh College, Jeddah P.O. Box 23323, Saudi Arabia; 3 Department of General Surgery, Dr. Soliman Fakeeh Hospital, Jeddah P.O. Box 23323, Saudi Arabia; leenahatem0987@gmail.com; 4 College of Medicine, Dr. Soliman Fakeeh College, Jeddah P.O. Box 23323, Saudi Arabia; 19000067@fcms.edu.sa (R.E.B.); ola.z2000@outlook.sa (O.A.Z.)

**Keywords:** tissue expander, burn, scar, review article, the patient and observer scar assessment scale (POSAS), Wound-QoL-17

## Abstract

Tissue expansion (TE) is a rather widely accepted technique in plastic surgery, with variable indications; among numerous other indications, it is commonly used in scar management. In our research, we focused on the significance of TE as an important armamentarium and valid solution for the treatment of large and old post-burn scars, with good cosmetic and functional outcomes. Our study is a retrospective analysis conducted at Dr. Soliman Fakeeh Hospital, Jeddah, during the period from 2020 to 2023. This retrospective analysis included 15 tissue expanders, which were inserted in eight patients with large post-burn scars. Each patient underwent two stages of scar repair using variable-size tissue expanders. Post-operative assessment for scar quality and patient satisfaction was conducted using the POSAS score system in addition to standard documentation. Our study sheds light on the satisfactory and accepted cosmetic and functional outcomes for skin tissue expanders for post-burn scars and deformities.

## 1. Introduction

Tissue expansion (TE) is a well-established and important technique in the field of plastic surgery, offering a versatile approach to tissue cover. It has significantly impacted the way procedures are approached and has become a valuable tool in the plastic surgery toolkit. While it is not a contemporary method, it remains accessible and holds significant clinical relevance [1]. It is a relatively simple procedure that enables the body to “grow” extra skin for use in reconstructing almost any area of the body. A silicone balloon expander is inserted under the skin, near the area to be repaired, and then gradually filled with saline or carbon dioxide over time, thereby causing the skin to stretch and grow [1]. The popular tissue expanders offered by manufacturing companies are usually circular, rectangular, or crescentic (croissant) in shape and are usually produced in commonly required volumes or capacities, which range from 50 cc to 1000 cc in increments of 50 cc to 100 cc [2]. The first reported case was by Neumann in 1957 [1]. He inflated the skin above the ear to cover the cartilage graft needed for the reconstruction of an avulsed ear. Radovan used TE in 68 breast reconstruction patients almost 20 years later [3]. The concept of expanders was basically that in pregnant women, the breast and abdominal wall enlarge many times, but the thickness and skin appendages are preserved. A fresh opportunity was created by Radovan’s invention [3]. Since then, TE has become an essential component used for the reconstruction of numerous congenital and acquired wounds in both children and adults [4]. There is an injection site where saline can be administered to inflate the expander, and it is made of a flexible and resilient silicone substance [4].

Burns are defined as injuries to the skin and underlying tissues caused by heat, chemicals, electricity, or radiation [5]. The cellular mechanisms involved in burn injuries are complex and multifaceted. Inflammation plays a crucial role in the pathophysiology of burns, with the release of pro-inflammatory mediators and the recruitment of immune cells to the site of injury [5]. The inflammatory response can have both beneficial and detrimental effects, as it is necessary for wound healing but can also contribute to tissue damage and organ dysfunction [5]. One cellular mechanism of interest in burn injuries is cell death, specifically necrosis and apoptosis. Necrosis is the predominant mechanism of cell death in burn injury progression, particularly in the early stages [5]. Apoptosis, on the other hand, appears to play a role in the interface between necrotic and viable tissue. Understanding the relative degrees and rates of necrosis and apoptosis is important for elucidating the progression of burn injuries [5].

There is a strong interest in clinical practice to improve the treatment and outcomes of burn patients. Burn injuries are devastating and can lead to severe metabolic stress, inflammation, and impaired immune function [6]. The use of glutamine (GLN) as a therapeutic intervention in burn injuries has shown promising results in reducing gram-negative bacteremia and improving outcomes. Additionally, cell therapy using mesenchymal stem cells (MSCs) has shown potential in promoting wound healing and reducing inflammation in burn injuries [7]. Bacteriophage-based therapy is also being explored as a potential treatment for burn wound infections [8].

Burns are a rather prevalent problem throughout the world, predominantly affecting low to middle income countries [5]. Burns are a prevalent health concern in Saudi Arabia, with a significant impact on the younger population. Children constitute a notable majority, accounting for 52% of all reported burn cases. Additionally, there is a gender disparity, as males outnumber females with an overall ratio of 1.42:1 in burn incidents [9]. In cases of acute burns, after the immediate risk to life is reduced, there may be physical and psychological issues caused by unsightly and dysfunctional scars. A number of methods, including the use of expanders, are available for the repair of burn-related scars. Most often, a combination of techniques is required and these are usually applied in numerous surgical procedures. In large scars, other scar management techniques are of limited value in comparison to TE due to cost, longevity of the treatment period, and even limited improvement. This study aims to review previous studies on the use of biological expanders in post-burn patients and demonstrate our experience with tissue expanders as a versatile technique for significant cosmetic improvement in large post-burn scars treated at Dr. Soliman Fakeeh Hospital (DSFH). It also describes a new surgical technique utilized at DFSH. Furthermore, the study aims to reveal postoperative complication rates and assess the cosmetic and functional outcomes of surgery.

## 2. Materials and Methods

### 2.1. Data Collection and Patient Selection

**Figure 1:**
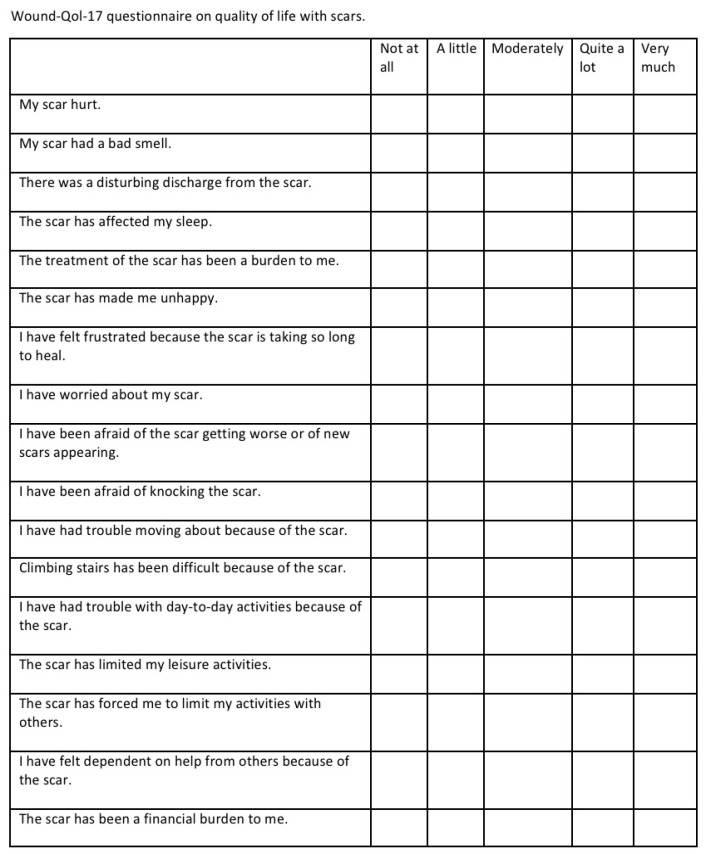
Wound-Qol-17 questionnaire on quality of life with scars.

**Figure 2:**
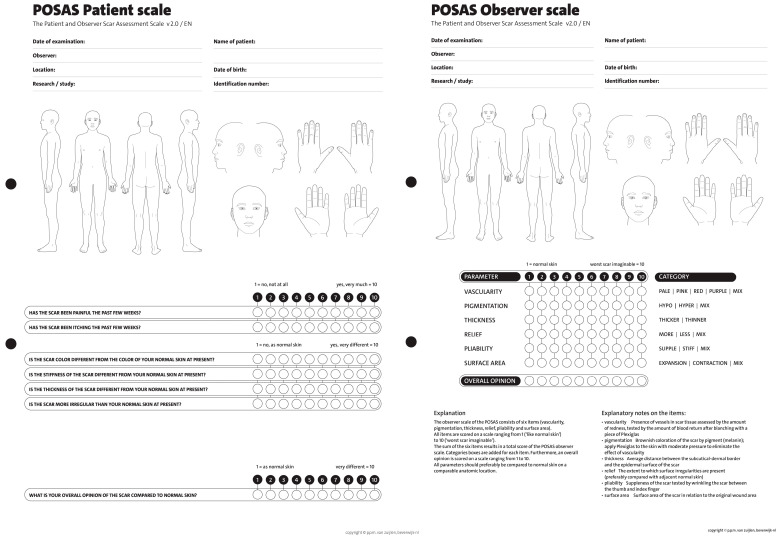
The Patient and Observer Scar Assessment Scale (POSAS).

**Figure 3:**
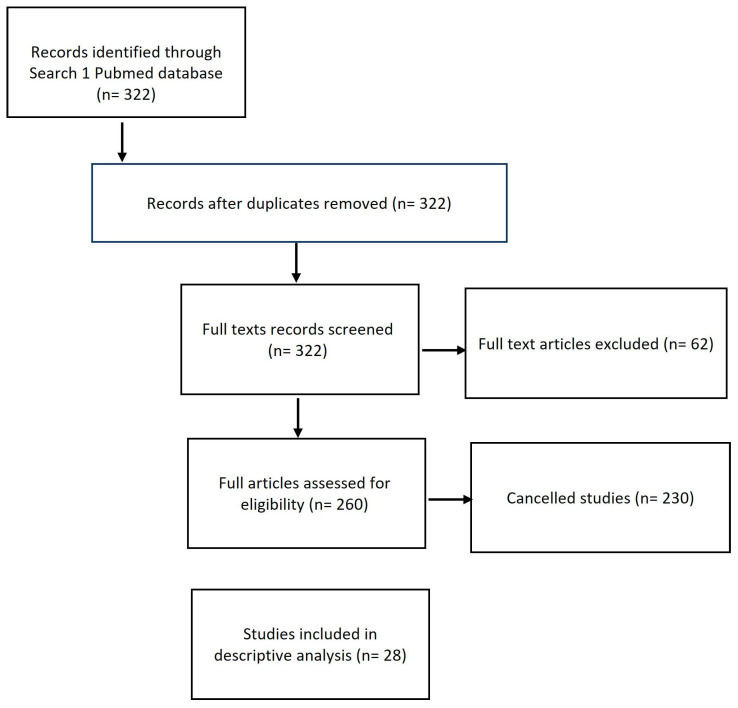
Prisma flow chart.

Ethical approval was obtained from the DSFH Institutional Review Board (Approval no. 312/IRB/2022). This study is a retrospective analysis of 15 tissue expanders inserted in eight patients in order to perform scar reconstruction and repair. Demographic data, biological tissue expander device type, volume of devices, site of insertion of the tissue expander, our technique for tissue expander insertion, complications, and outcome were collected. All tissue expanders were rigid bases with remote ports and self-filling. All data have been collected in a special questionnaire, and statistical analysis was performed using SPSS 21 software. In addition, a questionnaire was administered to patients in a clinic sitting before insertion of the tissue expanders to determine satisfaction using the Wound-QoL-17 questionnaire on Health-related Quality of Life in Chronic Wounds (13). The questionnaire was adjusted to fit our objective, where the term “wound” was changed to “scar” (Figure 1). The scar was assessed postoperatively using the Patient and Observer Scar Assessment Scale (POSAS) in the clinic by the primary surgeon (14). The scale is widely used for measurements of scar quality and was used for measuring satisfaction in our study by the patient as well as the surgeon by rating the quality of the scar (Figure 2). We searched the PubMed data engine using the keywords “burns”, “tissue expander”, and “biological tissue expanders”. The Prisma flow chart is depicted in the figure below in Figure 3.

### 2.2. Preoperative Preparation

In the clinic setting, we discussed the technique of the procedure as well as the advantages and disadvantages of the procedure with the patient. The normal skin surrounding the scar was examined, and we looked for the finest solution for expansion and skin restoration in the scar area. The most acceptable site for the surgeon was selected, which is always the nearby healthy skin so that the expander can be closer to the scar. Then, the size and type of tissue expanders were selected. In most of the cases, a rectangular or circular tissue expander was selected. The tissue expander was placed below the normal skin. We do not recommend placing the tissue expander beneath the scar since it may lead to complications. Placement of the tissue expander beneath the scar would likely cause skin necrosis, exposure of the tissue expander, and displacement of the tissue expander. 

### 2.3. First-Stage Procedure (Expander Implantation)

In the first procedure, a marking pen was used to mark the TE site, and a normal saline injection along with 1/100,000 epinephrine was administered. Then, the patient was prepped and draped in a similar manner. Prior to prepping and draping, it is crucial to administer epinephrine to ensure that the surgical site is bloodless and prepared for dissection. Thereafter, the incision was made tangentially, just on the border of the healthy skin and scar tissue. The incision length is typically half the length of the tissue expander. With Metzenblum scissors, we performed blunt dissection on the pocket. The size of the pocket was verified after the tissue expander was inserted. Following the removal of the TE, the pocket was packed with gauze soaked in a solution containing 1/100,000 epinephrine for hemostasis. Simultaneously, the TE was immersed in normal saline along with antibiotics. A tunnel was created to facilitate the insertion of the port, which was positioned 5 cm away from the tissue expander and secured using a proline suture. Moreover, it was necessary that the port be placed away from the tissue expander so that it would not cover the port site after full expansion. Following the removal of the gauze, a vacuum drain was placed and fixed. Then, the tissue expander was installed in place and all tubes and drains were positioned underneath the tissue expander. Once all the air was completely expelled from the tissue expander, it was connected to the port. To ensure that the port and TE were closed and sealed, a few millimeters of normal saline were injected. The incision was closed in two layers, with interrupted Vicryl sutures, and the TE and port were both securely fastened in their sites. After closing the incision, the hemovac drain was connected and vacuumed. Then, a dressing was applied over the incision: a Tegaderm transparent film dressing was applied over the port site to allow better visualization and the injection of normal saline for subsequent expansion. Three weeks postoperatively, sutures were removed, and injections were administered weekly at a rate of 10 mL of normal saline per week. We usually wait three weeks postoperatively to begin expansion because the wound in the first three weeks is fragile, and any additional injection can disrupt the healing process and cause wound dehiscence.

During the postoperative period of 4–6 months, the administration of injections into the tissue expander will be continued until the desired size is achieved, typically coinciding with the occurrence of the second operation. Our intended flap size is conventionally set to be 2 cm longer than the required flap length, thereby providing the surgeon with an additional 2 cm of tissue in case of any unforeseen complications during the subsequent corrective surgery. This approach ensures the safety and complete removal of scar tissue.

### 2.4. Second-Stage Procedure (Removal of the Tissue Expander and Scar Revision)

In the second procedure, after prepping and draping, the required flap is fashioned, and the incision site is marked with a marking pen. Thereafter, a solution of regular saline and 1/100,000 epinephrine is injected into the scar tissue. The incision used to remove the TE is the same one used to insert it. This is a critical aspect. During the first operation, the surgeon must choose the type of flap he will use for reconstruction as well as the incision he will need for the second procedure. Advancement flaps, rotational flaps, bilobed flaps, premade flaps, and transpositional flaps can all be created with a tissue expander. The type of flap is selected depending on the nature of the scar as well as the availability of normal tissue around the scar and the flexibility of the normal tissue that will be transplanted. This preparation is, in fact, part of the first stage operation, and we always have to discuss with the patient the best possible flap option after expander removal. In most cases, the transposition flap is longer than the advancement flap; it can also be used to remove a longer scar. For the majority of our patients, we do not remove the entire capsule. Occasionally, a partial release in the center of the flap can help with a wider arc of rotation or advancement. Overexpansion rarely solves the problem and allows the flap to extend to the appropriate length without partial release.

Next, the expanded tissue was used to form and cut the desired flap, which was then used to cover the scar. Interim staples were utilized to hold the flap in place, serving as a temporary measure to approximate and secure the flap without exerting undue pressure on the surrounding skin. These staples were subsequently removed to ensure optimal healing and minimize any potential impact on the skin. Then, a marker was used to outline the edge of the flap, and the staples were taken out; thereafter, the flap was reinserted into its original position. After hemostasis, the identified scar tissue was removed, and the flap was repositioned and sutured in two layers using Vicryl and Monocryl (or nylon) sutures; a majority of the time, a vacuum drain is required for this. Once the circulation of the flap was verified, a dressing was applied. Throughout the first and second postoperative days, the flap was continuously examined for signs of poor circulation, necrosis, or any other abnormalities. In case something untoward happens, the surgeon will take the stitches out and reduce the tension on the flap. Additionally, it is possible that the flap could be resistant to ischemia and new circulation for two or three days while it is covered with a dressing. After one or two days, the flap can be re-sutured to the area. Because the flap has already acclimated to the new circulation, there will likely be no necrosis at this point. The drain is removed after the daily discharge is less than 10 cc–15 cc, which generally takes one or two days. After seven to eight days, the sutures are removed, if necessary. We advise the patient to mobilize as early as possible to avoid deep vein thrombosis and other complications. This may range from patient to patient depending on the extent of surgery. We recommend early mobilization from the first day. Nevertheless, patients are advised to perform gradual mobilization in order not to make any breakdowns to the wounds. Patient were able to fully mobilize after one to two months of insertion of the tissue expanders, similarly to full mobilization after removal of the tissue expander and reconstruction of the old scar. The expectant restoration of full and normal mobilization after the whole process of insertion and removal, along with the period of filling was between six months to one year in patients of this study.

### 2.5. Outcome

The outcome was measured according to two parameters: cosmetic and reconstructive. Preoperatively, the cosmetic outcome was mainly measured by the patient’s satisfaction with the scar using the Wound-QoL-17 questionnaire. Postoperatively, the reconstructive outcome was assessed using POSAS by the primary surgeon in the clinic.

### 2.6. Data Analysis 

After the data were coded, revised, and filtered, SPSS ver. 21 (Chicago, IL, USA) was utilized for data analysis. Descriptive statistics were used; frequency and proportions were used for qualitative variables (individual answers for patient satisfaction), while means and standard deviations (SDs) were used for quantitative variables (satisfaction score and scar assessment).

We calculated the mean of the results by calculating not at all (1 point), a little (2 points), moderately (3 points), quite a lot (4 points), and very much (5 points). 

## 3. Results

### 3.1. Data

#### 3.1.1. Demographic Data

Our results revealed that all our patients were female (100%). The mean age was 27 years (range 22–32 years). In addition, the indication of a tissue expander was a burn (100%).

#### 3.1.2. Biological Tissue Expander Device Type

A rectangular-shaped tissue expander was used in this study.

#### 3.1.3. Volume of Device

The device was filled with normal saline (10 mL) after three weeks of placement of the tissue expander.

#### 3.1.4. Site of the TE Expander

Most scars were in the extremities (right thigh (40%), left thigh (30%)), followed by the right (20%) and left (10%) abdomen (Table 1).

#### 3.1.5. Complications

No complications were found in the postoperative period.

### 3.2. Results of Patients’ Satisfaction and Scar Assessment

#### 3.2.1. Patients’ Satisfaction (before Reconstruction with an Expander)

**Figure 4:**
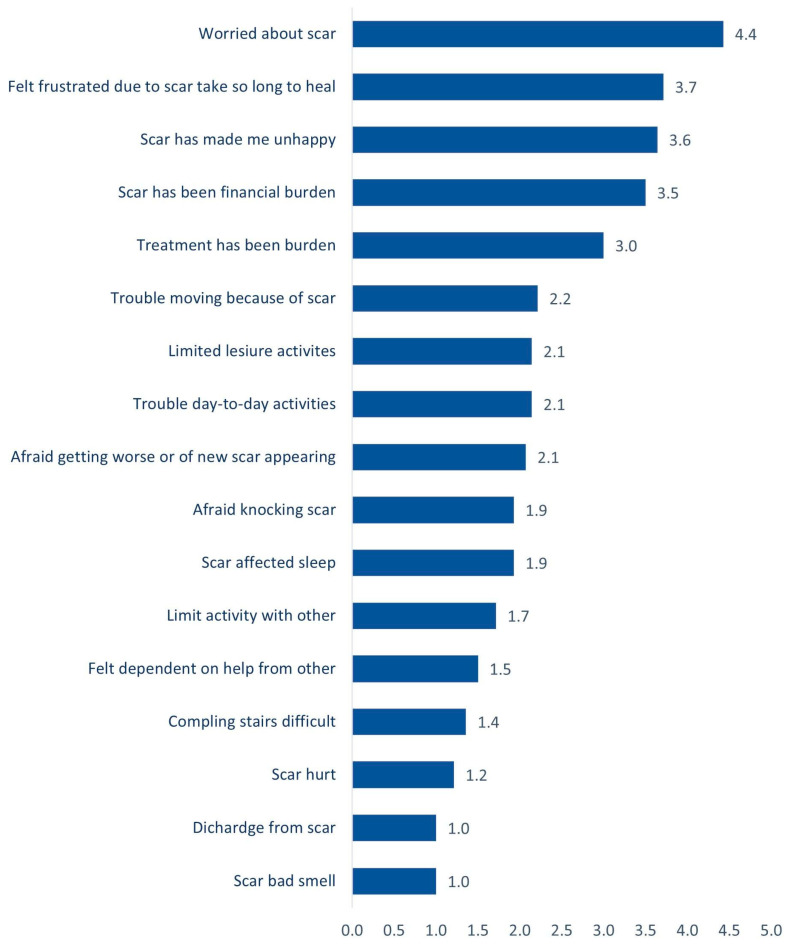
Patient’s satisfaction average score on Wound-QoL-17 items before surgery.

The Wound-QoL-17 questionnaire was distributed to patients before the operation. The questionnaire contained 17 items and each question had five options (not at all, a little, moderately, quite a lot, very much). The results are presented in Table 2 and Figure 4.

Table 2 illustrates patients’ satisfaction before surgery. All cases were worried to different degrees: 92.9% said that treatment has been a burden, 85.7% said that scars have made them unhappy, 85.7% were frustrated because scars take so long to heal, and 78.6% said that scars have been a financial burden. Approximately 42.9% had trouble moving because of scars, and 42.9% had trouble with day-to-day activities. These statements were related to the post-operative surgical scar. None of the patients experienced a bad smell or discharge from the scar. Figure 4 presents the patients’ average satisfaction score for Wound-QoL-17 items before surgery. The highest mean (low satisfaction) score was for being worried about scars (4.4 out of 5); this was followed by being frustrated due to scars taking so long to heal (3.7 out of 5), being unhappy (3.6 out of 5), financial burden due to scars (3.5 out of 5), treatment burden of surgical scar post operatively (3.0 out of 5), trouble moving because of scars (2.2 out of 5), limited leisure activities (2.1 out of 5), and trouble with day-to-day activities (2.1 out of 5). The lowest score was for discharge and smell (1 out of 5 each). Respondents in our study referred to the pre-operative scar as the “post-burn scar”, which typically represents a wide scar. Conversely, the post-operative scar was described as the resultant scar after reconstruction with pre-expanded tissue flaps.

#### 3.2.2. Scar Assessment (after Reconstruction with an Expander)

**Figure 5:**
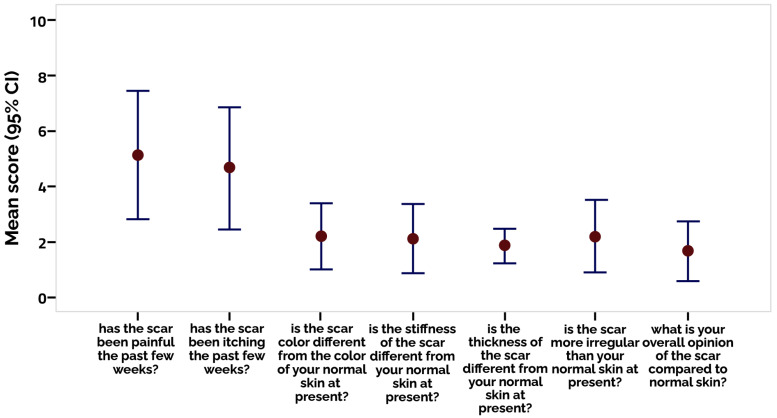
Post-operative scar assessment using POSAS observer scale among study patients.

**Figure 6:**
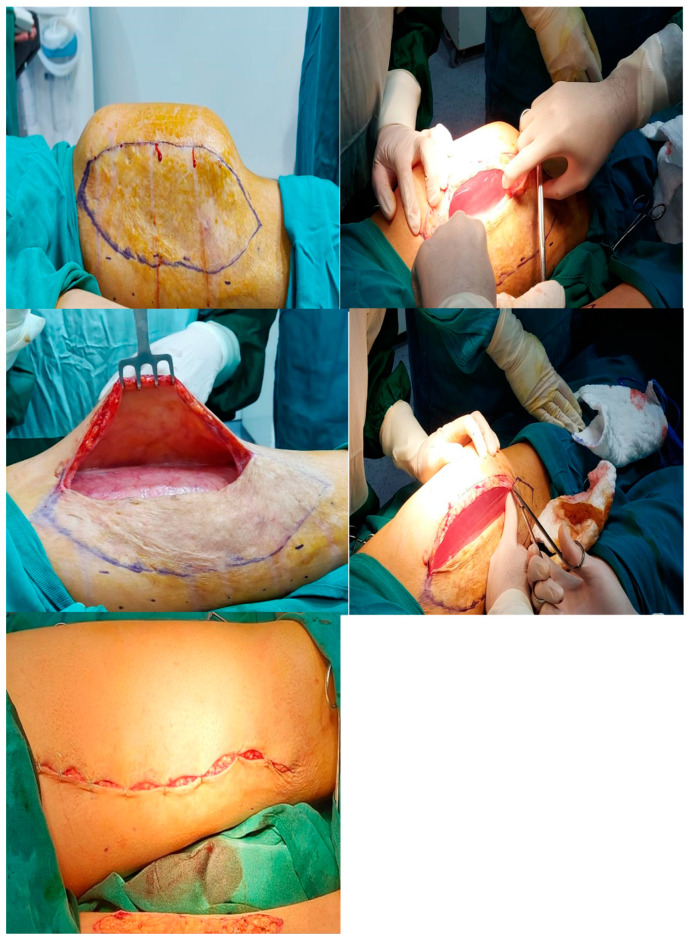
Tissue expander in the thigh.

**Figure 7:**
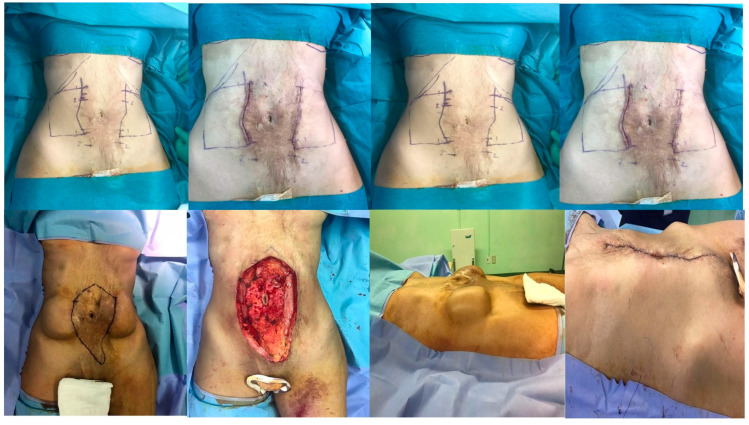
Tissue expander in the abdomen.

The following section is related to the postoperative surgical scar. Seven questions regarding satisfaction with the scar were answered by all eight patients, and a total of 15 tissue expanders are presented in Table 3. The scores ranged from 1: no, not all, normal to 10: yes, very much, very different. The highest mean score was for a painful scar during the last few weeks (5.1 out of 10), an itchy scar (4.7 out of 10), a color different from the color of normal skin at present (2.2 out of 10), a scar more irregular than your normal skin at present (2.2 out of 10), scar stiffness (2.1 out of 10), and scar thickness (1.9 out of 10). The lowest average score was for the patients’ overall opinion of the scar compared to normal skin (1.7 out of 10) (Figure 5). It was noted that prosthetic exposure was the highest complication postoperatively by a percentage of 13.81% (Table 4). We have shared in this paper some figures of tissue expanders in the thigh and abdomen, respectively (Figure 6 and Figure 7).

## 4. Discussion

Tissue expanders are one of the most frequently used implants in cosmetic and reconstructive surgery [10]. In this study, we found that tissue expander use is linked with good patient satisfaction post-operatively, good surgery outcome, and minor complication rates. (Figure 1, Figure 2 and Figure 3 and Table 4). Patient satisfaction is a crucial aspect of our study, and we have assessed it through the patients’ responses to our survey. Additionally, we have compared the status pre- and post-operatively to evaluate the overall patient experience. Furthermore, the objective evaluation of the surgeon regarding the wound itself and the low complication rate associated with this surgical procedure also contributed to the high level of satisfaction observed. In particular, the exposure of tissue expanders (TE) in 13% of patients is considered a low complication rate compared to other reconstructive surgeries. This can be attributed to the preservation of healthy tissue in most cases, even after the removal of the expanders. Both functional outcomes and cosmetic results have been assessed, further highlighting the positive outcomes of the surgery.

As mentioned in Figure 3, we reviewed previous studies and collected parameters (number of participants, age mean, indication, complications (seroma, hematoma, infection), outcomes (wound dehiscence, cosmetic, functional, aesthetic/reconstructive), and failure rate. Interestingly, we found a minor number of complications in hematoma, seroma, and infection. Other specific complications have been mentioned in other studies, such as skin flap necrosis and extrusion [11]. Both expander capacity and anatomical location had an impact on the failure rate, where a failure rate reaching 70% of our expanders was in the lower extremities. TE, like any other surgical operation, is accompanied by postoperative problems. To improve surgical outcomes, it is essential to conduct thorough assessments of problems that arise during TE [11]. We reviewed and compared our data to prior retrospective research using one database (PubMed). The rates of complication or failure of expander therapy for burn patients were not always directly mentioned, but were measured from data presented in research articles, which limits direct comparison due to different definitions of rates and complications (e.g., major or mild complication vs. early and late complications).

In our study, we correlated the outcome of tissue expanders with different outcomes. We measured patients’ satisfaction with scars preoperatively using the Wound-qol-17 questionnaire on health-related quality of life in chronic wounds. This revealed that most patients’ scars did not hurt, did not small bad, or have a disturbing discharge. Patients felt frustrated because the scar took so long to heal and worried about the scar moderately, with a mean of 4.43. Additionally, we evaluated scar outcome postoperatively using the POSAS observer scale by the primary surgeon. We used the best possible survey according to the stage, whether it is pre- or post-operative. Thus, we chose a specific survey pre- and post-operatively. The average of the results was measured and revealed that most patients had a painful scar, and it was itchy, with an average score of 4.7 and 5.1, respectively. The supposition of the scar on ordinary skin was normal in all patients.

Gender, age, tobacco use, and repeated expander implantation were not reported to increase the incidence of complications [12]. In the current study, complications of prosthesis were detected in five patients (13.814%), including prosthesis exposure (5.56%), prosthesis site infection (2.78%), leakage of the prosthesis (2.78%), hematoma (2.78%), and necrosis of the injection port site (2.78%). Other studies reported infection rates of 6% and 8.25% [1,13].

Further, our study reported no complications and revealed that most patients were satisfied with the results of the operation. Infection, which is one of the major complications of TE, may result in premature removal and even failure [1,14]. TE is frequently used in reconstructive surgery. Although the surgical procedure is typically considered simple, reported complication rates of TEs can reach up to around 40% [15]. In contrast, our study reported no complications. Based on our above-mentioned findings, our primary focus for patient education is to enhance their understanding of skin expanders and the two-stage nature of the procedure. It is crucial for patients to be well-informed about each step of the surgery and the expected outcomes. We aim to reassure patients that the ultimate result of skin reconstruction using expanded tissue flaps can be more satisfactory compared to the initial scar.

Our study had two main limitations that should be acknowledged. Firstly, all scars treated were excised in full or completely, focusing on patients who presented with scars remaining after tissue expansion surgery. This selection criteria may limit the generalizability of our findings to scars resulting from other types of surgeries or injuries. Secondly, the inclusion of different body parts in our study might have introduced heterogeneity in the results. We suggest that future researchers conduct comparative studies with a similar design but specifically focus on upper limb, lower limb, trunk, or breast scars. This approach would provide more specific and targeted insights, contributing to the existing literature and expanding our knowledge in these specific areas.

To conclude, using biological TE with the best practice technique as demonstrated in our study is rewarding for both patients and surgeons. We correlated biological TE with patients’ satisfaction and quality of life. We showed that it was significantly correlated and improved patients’ lives. Thus, we believe that using biological tissue expanders can be strongly considered as standard for care of scars with nearby healthy skin, where expansion can provide a big amount of viable tissue that can replace the scar completely. The effectiveness of the results from TE may be increased with knowledge of the most frequent complications, appropriate training of medical staff, and thorough and ongoing patient education.

## 5. Conclusions

In our study, we emphasize that TE is an effective technique for improving cosmetic and functional outcomes in large burn scars, with a superior satisfactory result in comparison to other modalities for large burn scars.

## Figures and Tables

**Table 1:** Site of tissue expander.

Site	Number
Breast	1
Rt abdomen	2
Lt abdomen	1
Rt thigh	4
Lt thigh	5
Rt arm	1
Lt arm	1

**Table 2:** Patients’ satisfaction using “Wound-QoL-17” questionnaire (before reconstruction with expander).

Wound-QoL-17 Items	Not at All		Little		Moderate		Quite a Lot		Very Much		Mean (SD)
	No	%	No	%	No	%	No	%	No	%	
Pain from the scar	11	78.6%	3	21.4%	0	0.0%	0	0.0%	0	0.0%	1.21 (0.43)
Bad smell from the scar	14	100.0%	0	0.0%	0	0.0%	0	0.0%	0	0.0%	1.0 (0.0)
Discharge from the scar	14	100.0%	0	0.0%	0	0.0%	0	0.0%	0	0.0%	1.0 (0.0)
Scar affected sleep	10	71.4%	1	7.1%	0	0.0%	0	0.0%	3	21.4%	1.93 (1.69)
Treatment has been burdened	1	7.1%	2	14.3%	8	57.1%	2	14.3%	1	7.1%	3.0 (0.96)
Scar has made me unhappy	2	14.3%	0	0.0%	4	28.6%	3	21.4%	5	35.7%	3.64 (1.39)
Felt frustrated due to scar taking so long to heal	2	14.3%	0	0.0%	5	35.7%	0	0.0%	7	50.0%	3.71 (1.49)
Worried about scar	0	0.0%	1	7.1%	1	7.1%	3	21.4%	9	64.3%	4.43 (0.94)
Afraid of getting worse or of new scar appearing	9	64.3%	1	7.1%	1	7.1%	0	0.0%	3	21.4%	2.07 (1.69)
Afraid of injuring scar	10	71.4%	1	7.1%	0	0.0%	0	0.0%	3	21.4%	1.93 (1.69)
Trouble moving because of scar	8	57.1%	0	0.0%	3	21.4%	1	7.1%	2	14.3%	2.21 (1.58)
Difficulty in climbing stairs	12	85.7%	1	7.1%	0	0.0%	0	0.0%	1	7.1%	1.36 (1.08)
Trouble with day-to-day activities	8	57.1%	0	0.0%	4	28.6%	0	0.0%	2	14.3%	2.14 (1.51)
Limited leisure activities	8	57.1%	1	7.1%	2	14.3%	1	7.1%	2	14.3%	2.14 (1.56)
Limited activity with others	10	71.4%	2	14.3%	0	0.0%	0	0.0%	2	14.3%	1.71 (1.44)
Felt dependent on help from others	11	78.6%	1	7.1%	1	7.1%	0	0.0%	1	7.1%	1.50 (1.16)
Scar has been a financial burden	3	21.4%	2	14.3%	1	7.1%	1	7.1%	7	50.0%	3.50 (1.74)

**Table 3:** Scar assessment using the POSAS observer scale among study burn patients (after reconstruction with expander).

Post-Surgery Scar Assessment	Range	Mean	SD	Rank
Has the scar been painful the past few weeks?	1–10	5.1	4.2	1
Has the scar been itching the past few weeks?	1–10	4.7	4.0	2
Is the scar color different from the color of your normal skin at present?	1–7	2.2	2.1	3
Is the stiffness of the scar different from your normal skin at present?	1–8	2.1	2.3	5
Is the thickness of the scar different from your normal skin at present?	1–4	1.9	1.1	6
Is the scar more irregular than your normal skin at present?	1–10	2.2	2.3	4
What is your overall opinion of the scar compared to normal skin?	1–8	1.7	1.9	7

**Table 4:** Complications of prosthesis among studied burn patients.

Post-Surgery Prosthesis Complication	Percentage
Prosthesis exposure	13.81%
Prosthesis site exposure	5.56%
Prosthesis site infection	2.78%
Leakage of prosthesis	2.78%
Hematoma	2.78%
Necrosis of the injection port site	2.78%

## Data Availability

The data presented in this study are openly available.
